# Chicken IRF10 suppresses the cGAS-STING-IFN antiviral signaling pathway by targeting IRF7

**DOI:** 10.3389/fimmu.2026.1767491

**Published:** 2026-02-10

**Authors:** Sen Jiang, Jingyu He, Kun Wang, Mengjia Lv, Hongjian Han, Bingjie Sun, Nanhua Chen, Wanglong Zheng, Jianzhong Zhu

**Affiliations:** 1 College of Veterinary Medicine, Yangzhou University, Yangzhou, China; 2 Comparative Medicine Research Institute, Yangzhou University, Yangzhou, China; 3 Jiangsu Co-innovation Center for Prevention and Control of Important Animal Infectious Diseases and Zoonoses, Yangzhou University, Yangzhou, China; 4 Jiangsu Interdisciplinary Center for Zoonoses and Biosafety, Jiangsu Key Laboratory of Zoonosis, Yangzhou University, Yangzhou, China

**Keywords:** antiviral signaling, cGAS-STING, chicken, innate immunity, IRF10

## Abstract

**Introduction:**

The innate immune cyclic GMP-AMP synthase (cGAS)-stimulator of interferon genes (STING)-interferon (IFN) signaling axis is a cornerstone of antiviral innate immunity in chickens, with chicken interferon regulatory factor 7 (chIRF7) serving as the principal transcription factor downstream chSTING to initiate type I IFN production. Although the chicken interferon regulatory factor (IRF) family consists of eight members, the roles of those beyond chIRF7 in this pathway remain largely unexplored.

**Methods:**

Systematic screening was performed to analyze the roles of chicken IRF family members in the cGAS-STING-IFN antiviral signaling. Combined molecular biological and biochemical methods were utilized to explore the action mechanism of chIRF10 as a negative regulator of cGAS-STING-IFN antiviral signaling pathway in transfected cells and gene knockout cells, with or without different virus infections.

**Results:**

The chIRF10 was identified as a potent negative regulator of chicken cGAS-STING-IFN antiviral signaling. Mechanistically, chIRF10 mediated suppression required its IRF associated domain (IAD) but not its DNA binding domain (DBD). chIRF10 inhibited IFN activation triggered by cGAS-STING, as well as by the downstream kinases TBK1/IKKε and the transcription factor IRF7. Importantly, chIRF10 physically interacted with chIRF7 via its IAD, impairing chIRF7 dimerization and activation.

**Discussion:**

Collectively, these findings unveil a novel immunoregulatory function of chIRF10 and delineate a previously unrecognized mechanism fine-tuning the chicken cGAS-STING-IFN antiviral pathway, offering potential insights for developing interventions against avian viral diseases.

## Introduction

The innate immune system constitutes the host’s primary defense against invading pathogens, employing a network of pattern recognition receptors (PRRs), including Toll-like receptors (TLRs), C-type lectin receptors (CLRs), RIG-I-like receptors (RLRs), NOD-like receptors (NLRs), and cytosolic DNA sensors, to detect pathogen-associated molecular patterns (PAMPs) and damage-associated molecular patterns (DAMPs). This recognition triggers signaling cascades that culminate in the production of type I interferons (IFNs), cytokines, and chemokines, thereby mounting an initial antiviral response (1, 2).

Cyclic GMP-AMP synthase (cGAS) is a key cytosolic DNA sensor, which, upon binding double-stranded DNA (dsDNA), catalyzes the synthesis of 2′3′-cGAMP (3). cGAS detects cytosolic viral dsDNA as well as aberrant DNA originated from nucleus or mitochondria (4). The second messenger 2′3′-cGAMP activates the endoplasmic reticulum adaptor stimulator of interferon genes (STING), leading to the recruitment and phosphorylation of TBK1 and the transcription factor IRF3 (5–7). Phosphorylated IRF3 dimerizes, translocates to the nucleus, and initiates type I IFN transcription (8). STING can also activate NF-κB to augment inflammatory responses (9). Notably, chickens lack IRF3 and instead rely on IRF7 to mediate STING-driven IFN production (10, 11).

The IRF family of transcription factors plays pivotal roles in immune regulation, antiviral defense, and cell differentiation (12). While mammals possess nine IRF members, chickens encode a unique repertoire that includes IRF10 but lacks IRF3 and IRF9 (12, 13). All IRFs contain a highly conserved DNA-binding domain (DBD) at the N-termini, and all members except IRF6 contain a relatively conserved IRF-associated domain (IAD) at the C-termini (14). The DBD enables IRFs to bind specific interferon-stimulated response element (ISRE) promoter sequence (A/GNGAAANNGAAACT), whereas the IAD is for the formation of homodimers or heterodimers between IRFs or with other transcription factors, facilitating transcriptional activation or regulation (15).

Members of the IRF family play crucial roles in the regulation of IFN immune responses. IRF1, the first identified member, is constitutively expressed and can be induced by IFN−γ (16). It recognizes and binds to the ISRE promoter sequence, directly activating the transcription of type I IFNs and interferon-stimulated genes (ISGs) (17). IRF2 was identified through cross-hybridization with IRF1 cDNA (18), constitutively expressed and induced by viruses and IFNs (18). IRF2 acts as an antagonist of IRF1 by competing for binding to the promoter sequence (18). IRF3 serves as a key transcription factor in innate immune signaling pathways, activating type I IFN expressions (12). IRF7 shares high homology with IRF3 and can directly bind the ISRE promoter sequence (19). IRF5 and IRF6 are homologous proteins, with IRF5 but not IRF6 involved in the induction of IFN−α (12, 20). IRF9 is expressed in a variety of cells and a critical component of type I IFN signaling, forming the transcription complex ISGF3 with STAT1 and STAT2, activating the transcription of ISGs (21).

Chicken (ch) IRF family members are highly conserved with their mammalian counterparts but exhibit species-specific differences (10, 22). Although chicken interferon regulatory factor 7 (chIRF7) is established as the functional analog of mammalian IRF3 (10, 11), the contributions of other chIRFs, particularly chIRF10 present in chickens but absent in mammals, to innate immune cGAS-STING signaling remain poorly defined. Previous studies showed that chIRF10 is involved in the upregulation of two primary IFN-γ target genes, but interferes with the induction of the type I IFN target genes and serve as a negative regulator for host antiviral response (13, 23).

In this study, we systematically evaluated the involvement of chicken IRF family members in the cGAS-STING-IFN pathway. We confirmed that chIRF7 is the primary mediator of STING-induced IFN transcription and identified chIRF10 as a potent negative regulator. Further investigation revealed that chIRF10 interacts with chIRF7 via its IAD domain, suppressing IRF7 dimerization and activation. These results uncover a novel regulatory mechanism governing antiviral signaling in chickens and highlight chIRF10 as a key modulator of innate immunity.

## Materials and methods

### Antibodies and reagents

The FLAG Rabbit mAb (14793) and HA Rabbit mAb (3724) were acquired from Cell Signaling Technology (Boston, MA, USA). The mouse anti-FLAG mAb, mouse anti-HA mAb, mouse anti-GAPDH mAb, and mouse anti-GFP mAb were all from Transgen Biotech (Beijing, China). Mouse anti-Myc mAb (60003-2-Ig) was from ProteinTech (Wuhan, China). Anti-red fluorescence protein (RFP) mAb HRP-DirecT (M204-7) was from MBL Beijing Biotech (Beijing, China). Horseradish peroxidase (HRP) goat anti-rabbit IgG (H+L) highly cross-adsorbed secondary antibody and goat anti-mouse IgG (H+L) highly cross-adsorbed secondary antibody were all obtained from Sangon Biotech (Shanghai, China). Goat anti-mouse IgG H&L Alexa Fluor^®^594 (ab150120) were from Abcam (Cambridge, UK). Goat anti-rabbit IgG (H+L) cross-adsorbed 488 (35553) were from Thermo Fisher Scientific (Shanghai, China).

Lipofectamine 2000 was from Thermo Fisher (Sunnyvale, CA, USA). TransIT^®^-LT1 Transfection Reagent (MIR 2300) for HD11 was from Mirus Bio (Madison, WI, USA). TRIpure Reagent was from Aidlab (Beijing, China). Double-luciferase reporter assay kits, 2 × Phanta Max Master Mix (P515-01), HiScript^®^ 1st Strand cDNA Synthesis Kit, ChamQ Universal SYBR quantitative polymerase chain reaction (qPCR) Master Mix, and 2×Taq Master Mix (Dye plus) were all from Vazyme Biotech (Nanjing, China). The 2×MultiF Seamless Assembly Mix was from Abclonal (Wuhan, China). 2′3′-cGAMP and polydA:dT were from InvivoGen (Hong Kong, China). Protein A/G PLUS-Agarose was from Santa Cruz Biotechnology (sc-2003, CA, USA). 4′,6-Diamidino-2-phenylindole (DAPI) staining solution (C1005) was from Beyotime (Shanghai, China).

### Cells and viruses

The HEK-293T cells (ATCC Cat# CRL-3216) and chicken fibroblast DF-1 cells (ATCC Cat# CRL-12203) were maintained in DMEM (Hyclone Laboratories, USA) supplemented with 10% fetal bovine serum (FBS, Vazyme Biotech). The chicken macrophage HD11 cells (BCRJ-Linhagens celulares Cat# 0099) were cultured in RPMI 1640 medium (Hyclone Laboratories) supplemented with 10% FBS. Transfection was performed using the Lipofectamine 2000 or TransIT-LT1 following the manufacturer’s instructions. Newcastle disease virus (NDV)-RFP, avian influenza virus (AIV, H1N1), and vaccinia viruses (strains VACV and SMV) were all preserved in our laboratory.

### Plasmid construction and mutagenesis

The chicken (ch) cGAS, chSTING, chIRF7, and chIRF1 plasmids were previously constructed in our laboratory. The chIRF2 (NM_205196), chIRF4 (NM_204299), chIRF5 (NM_001031587), chIRF6 (XM_025143814), chIRF8 (NM_205416), chIRF10 (NM_204558), chTBK1 (NM_001199558), and chIKKϵ (XM_428036) open reading frames (ORFs) were amplified by RT-PCR from HD11 cells, using the designed primers as shown in **Supplementary Table 1**. The PCR products of chIRF were cloned into the *Bgl* II and *Kpn* I sites of pEGFP-C1 vector and the *EcoR* I and *Sal* I sites of p3×FLAG-CMV-7.1 vector, respectively. The PCR products of chTBK1 and chIKKϵ were cloned into the *EcoR* I and *EcoR V* sites of pCAGGS-2HA vector by seamless cloning. The mutation PCR primers of chIRF10 were designed and shown in **Supplementary Table 2**. The mutation PCR was performed with Phanta Max Master Mix and p3×FLAG-CMV-chIRF10 as the template, as we described previously (24).

### Luciferase reporter gene assay

Cells were transfected with IFN-β or ISRE luciferase reporters, a *Renilla* luciferase control, and expression plasmids as indicated. With or without further stimulation after transfection, luciferase activities were measured 24–48 h post-transfection using a dual-luciferase assay system, as we described previously (24).

### DNA and RNA extraction, reverse transcription, and quantitative PCR

Total cellular DNA was extracted using the HiPure Tissue DNA Mini kit (Magen, Guangzhou, China), and total RNA was extracted using TRIpure reagent following the manufacturer’s recommendations. The DNA and cDNA were used for measuring target gene expressions by qPCR with SYBR qPCR master Mix (Vazyme, Nanjing, China) using the 2^−ΔΔCT^ method after normalization to GAPDH mRNA levels. The sequences of qPCR primers are shown in **Supplementary Table 3**.

### Western blotting and co-immunoprecipitation analysis

The boiled and cleared cell lysate protein were run by sodium dodecyl sulfate polyacrylamide gel electrophoresis (SDS-PAGE) and then transferred to a PVDF membrane. The membrane was subjected to immunoblotting using the indicated primary antibodies and secondary antibodies, followed by detection with the ECL substrate and imaging system.

For co-immunoprecipitation (co-IP), the cleared cell lysates from the 6-well plate were incubated with 1 μg of specific antibody and Protein A/G PLUS-Agarose. The rinsed agarose was washed and eluted, and the immunoprecipitated protein in elution plus input controls were both subjected to Western blotting (WB).

### Native PAGE for chIRF7 dimerization

Cells from the 12-well plate were lysed in non-denaturing lysis buffer, and protein extracts were resolved on native polyacrylamide gels. chIRF7 dimers and monomers were detected by WB.

### Confocal microscopy

DF-1 cells transfected in a 15-mm glass bottom cell culture dish (5×10^5^ cells) were fixed, permeabilized, and stained with appropriate antibodies. Subcellular localization was visualized by laser-scanning confocal microscopy (LSCM, Leica SP8, Solms, Germany) at the excitation wavelengths 488 and 594 nm, respectively.

### Generation of chIRF10-knockout HD11 cells

CRISPR/Cas9-mediated knockout of chIRF10 in HD11 cells was achieved using specific gRNAs cloned into pX458-EGFP, as we described previously (24). Single-cell clones of IRF10^−/−^ HD11 cells were screened by genomic PCR and sequencing. Both gRNA encoding sequences and PCR primers are shown in **Supplementary Table 4**.

### Statistical analysis

Data are presented as mean ± SD from at least two independent experiments. Statistical significance was determined by Student’s *t*-test or analysis of variance (ANOVA) (*p < 0.05; **p < 0.01).

## Results

### Screening of chicken IRF family members for involvement in the chicken cGAS-STING-IFN signaling pathway

**Figure 1:**
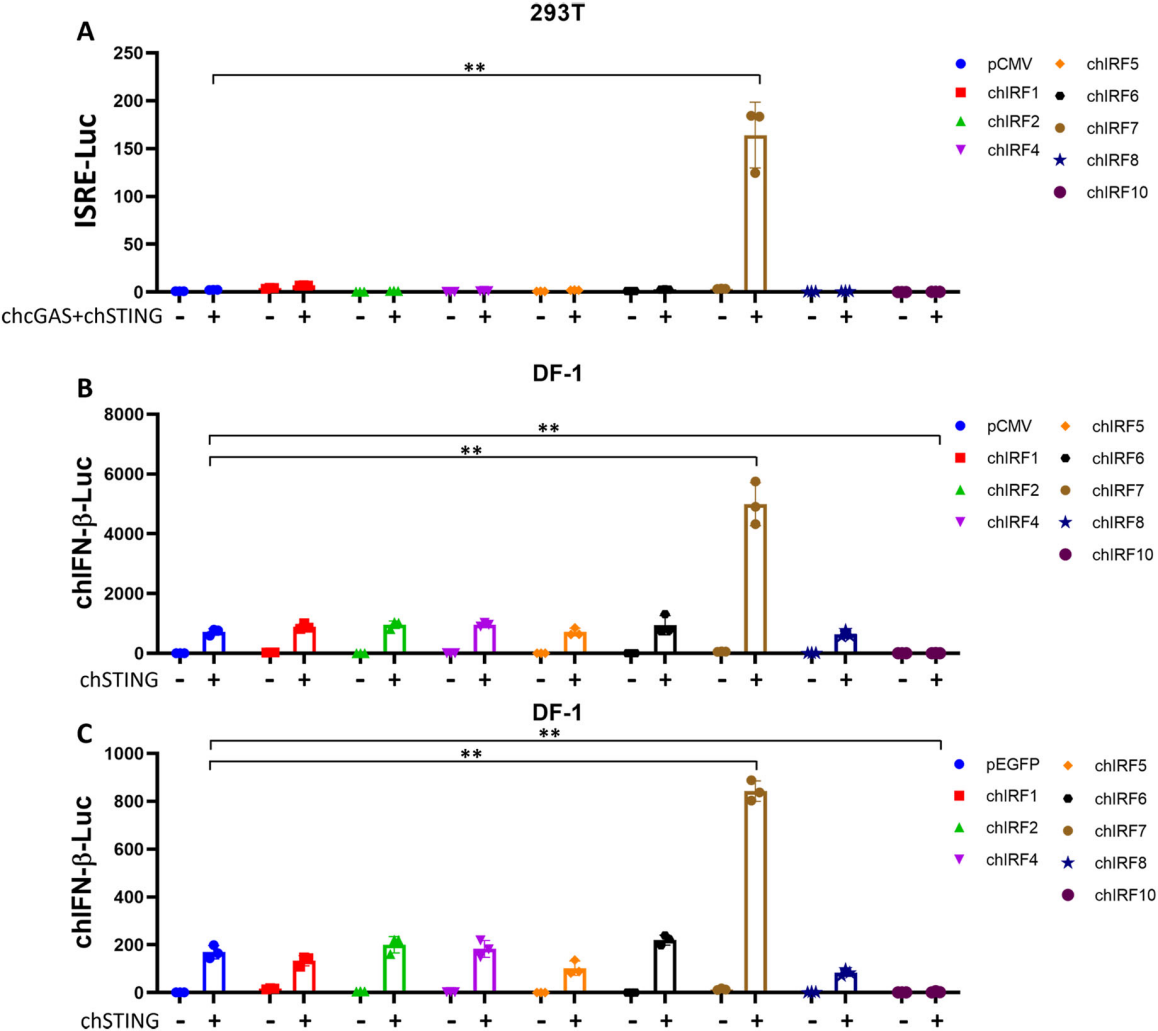
Analysis of chicken (ch) IRF family members in cGAS-STING-IFN signaling by promoter assays. **(A)** Flag-tagged chIRF family members were co-transfected with chGAS-STING into 293T cells, and ISRE promoter activity was measured at 24 h post-transfection. **(B)** Flag-tagged chIRF family members were co-transfected with chSTING into DF-1 cells, and chIFN-β promoter activity was measured at 24 h post-transfection. **(C)** GFP-tagged chIRF family members were co-transfected with chSTING into DF-1 cells, and chIFN-β promoter activity was measured at 24 h post-transfection. **p < 0.01 versus vector controls.

Previous studies including ours revealed that chicken naturally lack IRF3 and instead utilize IRF7 as the key transcription factor downstream of STING to activate type I IFN transcription (10, 11). However, the chicken IRF family comprises multiple members, and whether other IRFs participate in or regulate the chicken cGAS-STING-IFN antiviral signaling pathway remains unclear. To investigate this, we constructed expression vectors for eight members of the chIRF family including IRF1, IRF2, IRF4, IRF5, IRF6, IRF7, IRF8, and IRF10. Since 293T cells lack basal IRF7 expression, and chicken cGAS-STING cannot activate IFN signaling in this cell line (10), we therefore first conducted experiments in 293T cells and found that, among all chicken IRFs, only IRF7 synergistically activated the ISRE promoter in combination with chicken cGAS-STING (**Figure 1A**). Secondly, in the chicken fibroblast cell line DF-1 with basal IRF7 expression (11), chIRF7 enhanced chSTING-activated IFN-β promoter (**Figure 1B**), confirming the role of chIRF7 as a transcription factor mediating chSTING-activated IFN induction (10, 11). Furthermore, it was found that chIRF10 significantly suppresses chSTING-activated IFN-β promoter (**Figure 1B**). To validate these results, we repeated the experiment in DF-1 cells using pEGFP-C1-based IRFs other than p3×FLAG-CMV-expressed IRFs, and consistent results were yielded (**Figure 1C**). Together, these screening results suggested that among the chIRFs tested, only chIRF7 mediates IFN transcription upon activation of chicken cGAS-STING, confirming a previous finding. Notably, we identified chIRF10 as a significant inhibitor of the IFN signaling activated by chicken cGAS-STING.

### chIRF10 acts as a negative regulator of the chicken cGAS-STING-IFN signaling pathway

**Figure 2:**
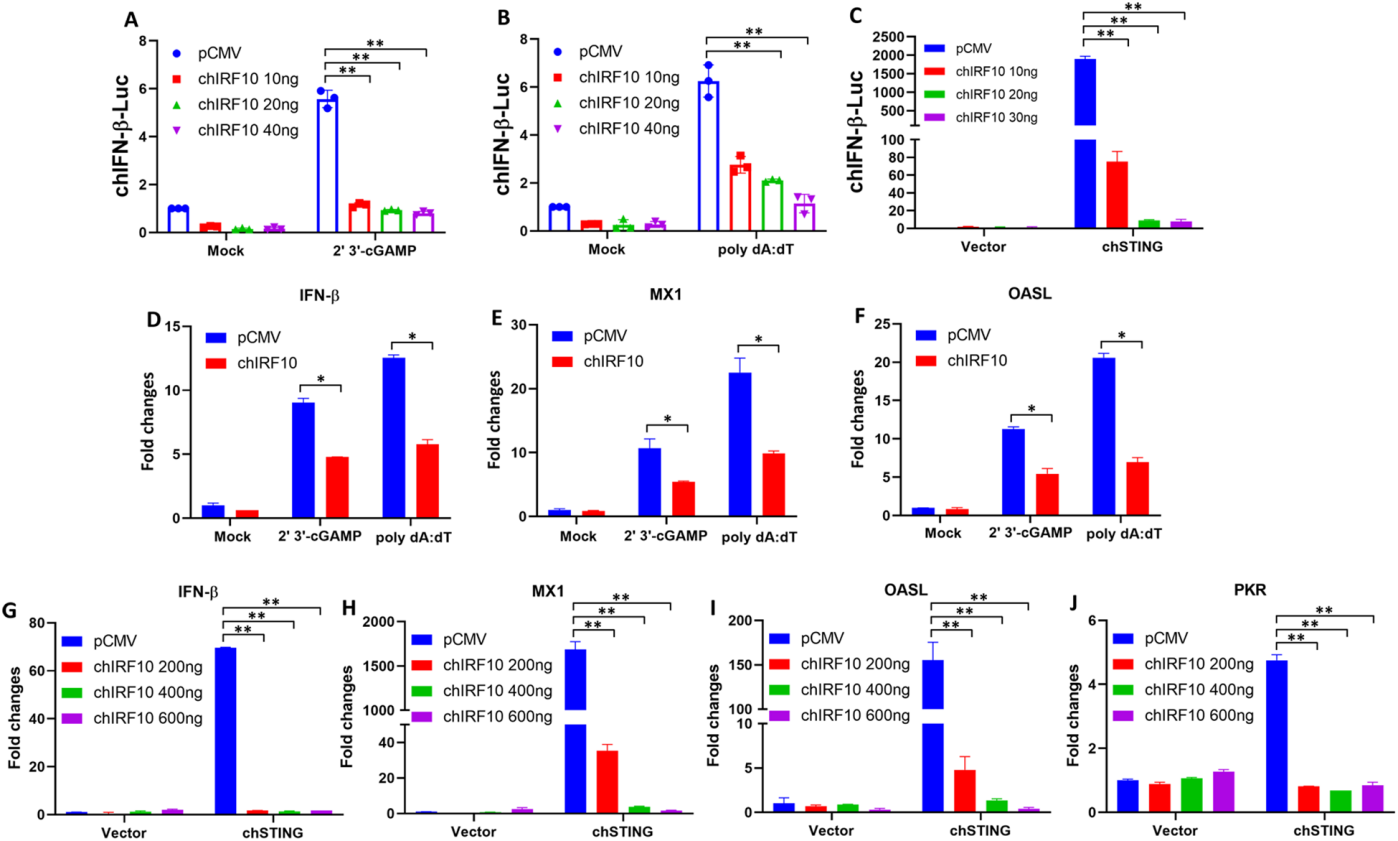
chIRF10 negatively regulates the chicken cGAS-STING-IFN signaling. **(A, B)** HD11 cells were transfected with increasing doses of chIRF10, which was normalized with pCMV vector. At 12 h post-transfection, the cells were stimulated with 2 μg/mL cGAMP **(A)** or 1 μg/mL poly dA:dT **(B)** for 12 h, followed by measurement of chIFN-β promoter activity. **(C)** DF-1 cells were co-transfected with chSTING and increasing doses of chIRF10, normalized with pCMV vector. Cells were collected at 24 h post-transfection to measure chIFN-β promoter activity. **(D**–**F)** HD11 cells were transfected with either chIRF10 or pCMV vector. At 12 h post-transfection, the cells were stimulated with cGAMP or poly dA:dT for 24 h, and the mRNA expression levels of downstream genes IFN-β **(D)**, MX1 **(E)**, and OASL **(F)** were detected by RT-qPCR. **(G**–**J)**)DF-1 cells were co-transfected with chSTING and increasing doses of chIRF10 for 48 h, and the mRNA expression levels of downstream genes IFN-β **(G)**, MX1 **(H)**, OASL **(I)**, and PKR **(J)** were measured by RT-qPCR. *p < 0.05 and **p < 0.01 versus vector controls.

Subsequently, the negative regulatory function of chIRF10 was further characterized. Firstly, we used the chicken macrophage cell line HD11 and treated it with 2′3′-cGAMP (an agonist of STING) and poly dA:dT (an agonist of cGAS) to activate the cGAS-STING signaling pathway. The results showed that chIRF10 dose-dependently suppresses the activation of the chIFN-β promoter induced by both agonists (**Figures 2A, B**). Similarly, chIRF10 also inhibited chSTING-activated chIFN-β promoter in a dose-dependent manner in DF-1 cells (**Figure 2C**). Secondly, RT-qPCR results demonstrated that chIRF10 significantly reduces the mRNA expressions of downstream effector genes (IFN-β, MX1, and OASL) activated by either cGAMP or poly dA:dT in HD11 cells (**Figures 2D–F**). Thirdly, in DF-1 cells, chIRF10 dose-dependently inhibited the mRNA expressions of downstream antiviral genes (IFN-β, MX1, OASL, and PKR) activated by chSTING (**Figures 2G–J**). Collectively, these results further confirmed that chIRF10 acts as a negative regulator of the chicken cGAS-STING-IFN signaling pathway.

### chIRF10 inhibits the antiviral effect activated by chicken cGAS-STING signaling

**Figure 3:**
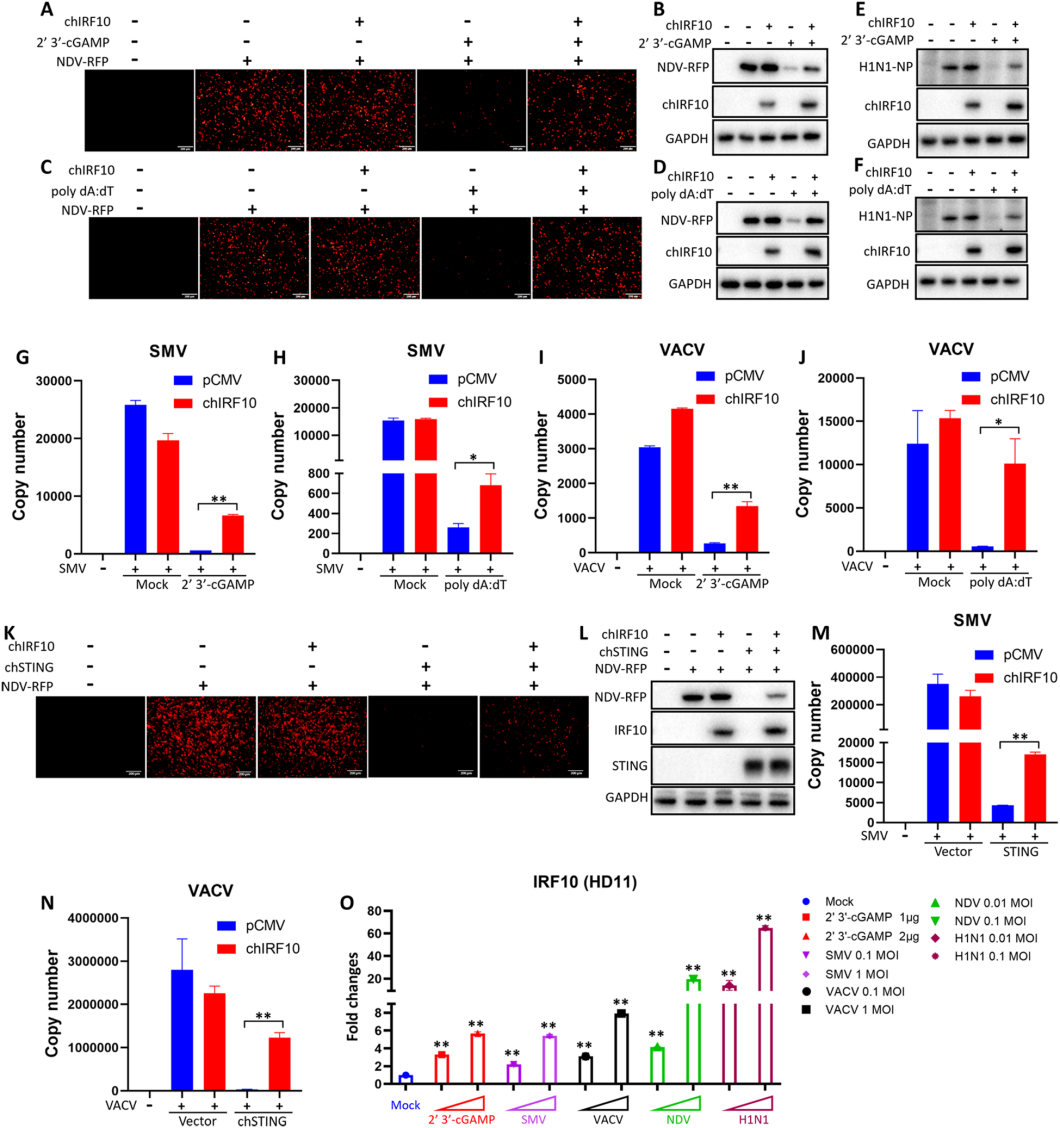
chIRF10 negatively regulates the antiviral function of chcGAS-STING signaling pathway. **(A–D)** HD11 cells were transfected with either chIRF10 or pCMV vector for 12 h, and then stimulated with cGAMP **(A, B)** or poly dA:dT **(C, D)** for 12 h, followed by infection with NDV-RFP at 0.01 MOI for 12 (h) RFP fluorescence was detected using fluorescence microscopy **(A, C)**, and cells were collected for WB analysis of RFP protein expression **(B, D)**. **(E, F)** HD11 cells were transfected and stimulated as in A, followed by infection with AIV (H1N1) at 0.01 MOI for 24 (h) The viral NP protein expression was detected by WB. **(G–J)** HD11 cells were transfected and stimulated as in A, followed by infection with SMV at 0.1 MOI for 24 h **(G, H)** or infection with VACV at 0.1 MOI for 24 h **(I, J)**. The viral copy number was measured by qPCR. **(K, L)** DF-1 cells were transfected with chIRF10 and chSTING as indicated for 24 h, and infected with NDV-RFP at 0.01 MOI for 12 h, followed by RFP fluorescence detection by fluorescence microscopy **(K)** and WB analysis of RFP protein expression **(L–N)** DF-1 cells were transfected as indicated for 24 h, and infected with SMV **(M)** or VACV **(N)** at 0.1 MOI for 24 h, followed by qPCR measurement of viral copy number. **(O)** HD11 cells were stimulated with cGAMP or infected with the corresponding viruses at the indicated doses. At 24 h post-infection, cells were collected and chIRF10 mRNA expression was detected by RT-qPCR. *p < 0.05 and **p < 0.01 versus controls.

Our previous studies have demonstrated that chicken cGAS-STING exerts broad-spectrum antiviral effects (25, 26). To investigate the impact of chIRF10 on the antiviral function of the cGAS-STING pathway, we utilized two RNA viruses, NDV and AIV (H1N1), along with two DNA viruses, vaccinia viruses SMV and VACV. The viral replication levels were determined as follows: NDV was observed for RFP, which it carries by engineering and detecting for RFP expression by WB. H1N1 was detected by WB for nucleoprotein (NP) expression. SMV and VACV were measured by qPCR for viral copy numbers. Results from infected HD11 cells showed that chIRF10 significantly inhibits the antiviral effects activated by cGAMP and poly dA:dT against NDV (**Figures 3A–D**), H1N1 (**Figures 3E, F**), SMV (**Figures 3G, H**), and VACV (**Figures 3I, J**). Similarly, in infected DF-1 cells, chIRF10 also suppressed the chSTING-activated antiviral activity against NDV (**Figures 3K, L**), SMV (**Figure 3M**), and VACV (**Figure 3N**). Finally, the mRNA expression level of chIRF10 following cGAMP stimulation and viral infections was measured. The results demonstrated a significant and dose-dependent upregulation of chIRF10 mRNA upon stimulation and various infections (**Figure 3O**).

### The negative regulatory activity of chIRF10 depends on its IRF-associated domain

**Figure 4:**
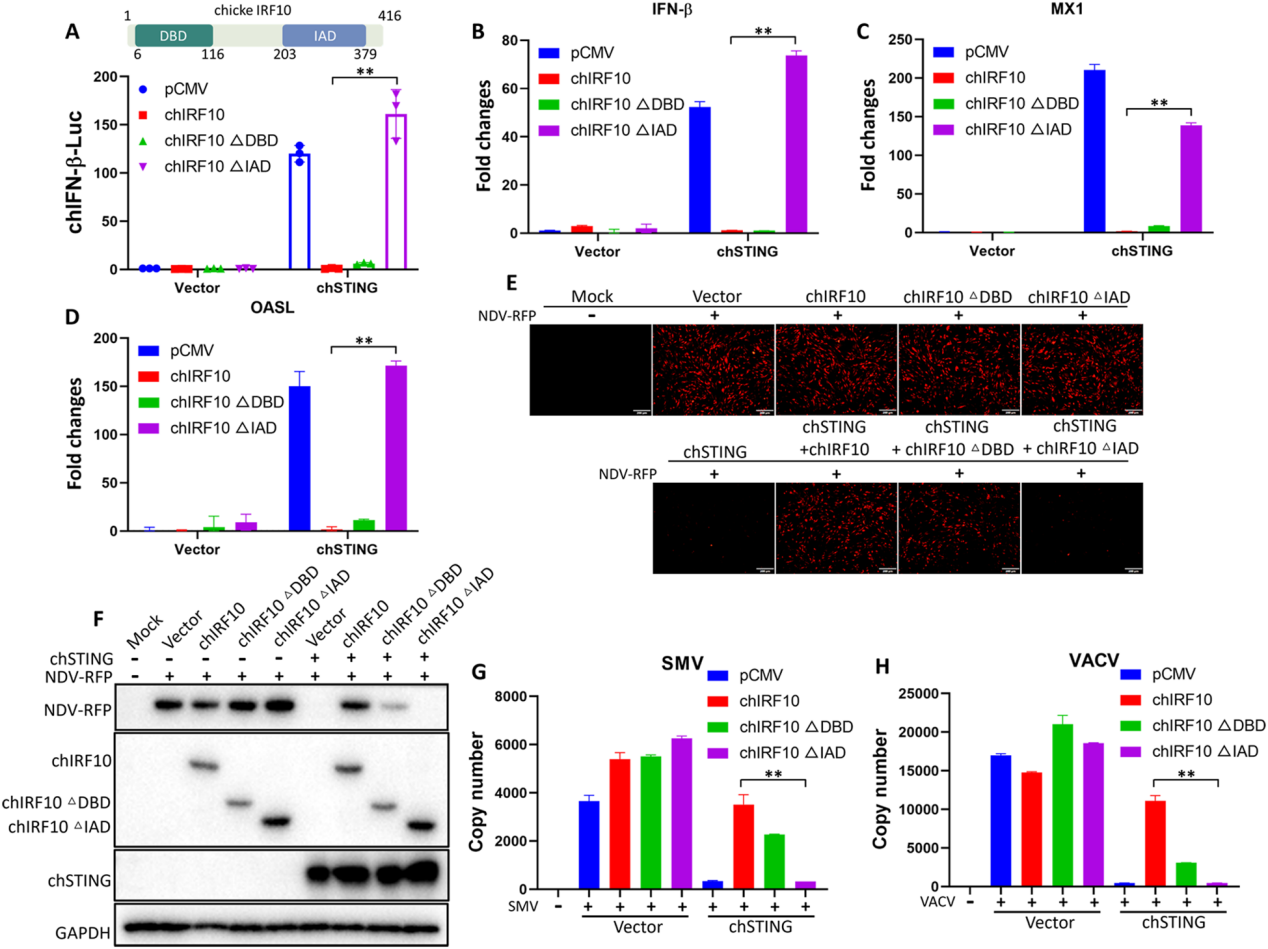
Deletion of the IAD, but not the DBD, abolishes the negative regulatory activity of chIRF10. **(A)** DF-1 cells were co-transfected with chIRF10 or its deletion mutants, with or without chSTING for 24 h, followed by measurement of chIFN-β promoter activity. **(B–D)** DF-1 cells were transfected as in A for 48 h, and the mRNA expressions of chSTING-activated downstream genes IFN-β **(B)**, MX1 **(C)**, and OASL **(D)** were detected by RT-qPCR. **(E, F)** DF-1 cells were transfected as indicated for 24 h and infected with NDV at 0.01 MOI for 12 (h) RFP fluorescence was observed using fluorescence microscopy **(E)** and RFP protein expression was detected by WB **(F–H)** DF-1 cells were transfected as indicated for 24 h and infected with SMV **(G)** or VACV **(H)** at 0.1 MOI for 24 (h) The viral copy number was measured by qPCR. **p < 0.01 versus controls.

The IRF family members contain two key domains: a DBD and an IAD. The DBD enables IRF to bind promoter DNA, while the IAD facilitates the formation of homodimers or heterodimers with itself or other proteins (27). The DBD of chIRF10 resides in amino acids 6–116, and its IAD is located within amino acids 203–379 (13) (**Figure 4A**). Accordingly, chIRF10 ΔDBD and ΔIAD mutants were constructed to investigate their effects on chSTING-activated IFN signaling and antiviral activity. Chicken IFNβ promoter assay results revealed that deletion of the IAD abolished chIRF10’s negative regulatory activity on chSTING-induced IFN signaling, whereas deletion of the DBD did not have such an effect (**Figure 4A**). Meanwhile, RT-qPCR results demonstrated that the deletion of the IAD abolished IRF10’s suppressive effect on the expressions of downstream genes IFN-β, MX1, and OASL following chSTING activation, while the deletion of the DBD had no impact (**Figure 4B–D**). Finally, antiviral assays demonstrated that chIRF10 ΔIAD had no impact on the antiviral capacity activated by chSTING against NDV (**Figures 4E, F**), SMV (**Figure 4G**), or VACV (**Figure 4H**). In contrast, chIRF10 ΔDBD still partially suppressed chSTING-mediated antiviral activity, although to a lesser extent (**Figures 4E–H**). Collectively, these results suggested that the negative regulatory function of chIRF10 depends on its IAD domain.

### chIRF10 suppresses IFN signaling activated by downstream mediators of chSTING, specifically chIRF7

**Figure 5:**
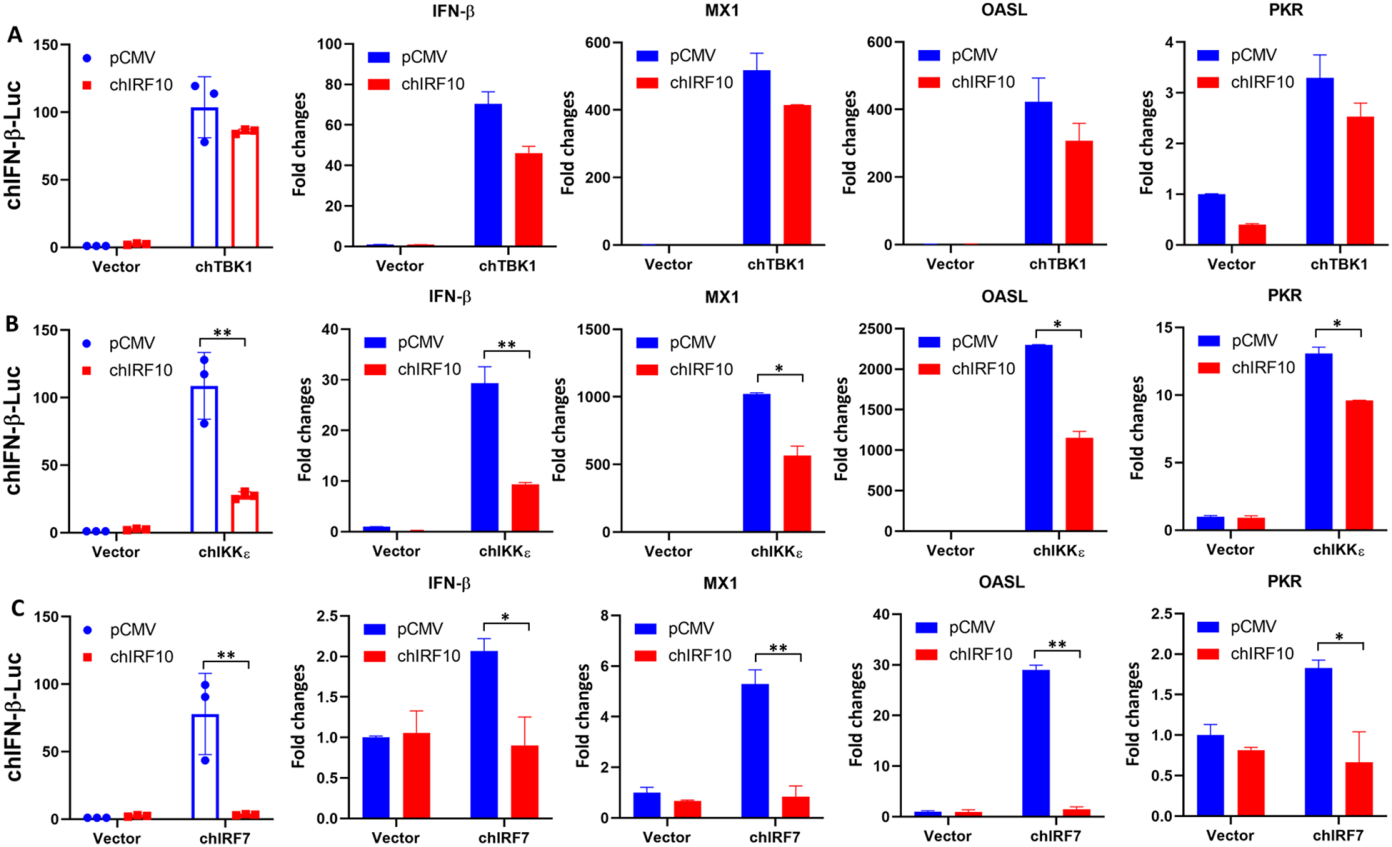
chIRF10 inhibits the IFN signaling activated by chTBK1, chIKKϵ, and chIRF7. **(A)** DF-1 cells were transfected with chIRF10 and chTBK1 as indicated. **(B)** DF-1 cells were transfected with chIRF10 and chIKKϵ as indicated. **(C)** DF-1 cells were transfected with chIRF10 and chIRF7 as indicated. chIFN-β promoter activity was measured at 24 h post-transfection, and the mRNA expression of downstream activated genes IFN-β, MX1, OASL, and PKR was detected by RT-qPCR at 48 h post-transfection. *p < 0.05 and **p < 0.01 versus controls.

TBK1 and IKKϵ are crucial protein kinases downstream of STING, while chIRF7 is a key transcription factor downstream of chSTING (10, 28). Therefore, expression plasmids for chTBK1 and chIKKϵ were constructed to examine the effect of chIRF10 on their activation of IFN signaling. The results showed that chIRF10 slightly downregulated the IFN-β promoter activity activated by chTBK1, as well as the mRNA expressions of downstream genes including IFN-β, MX1, OASL, and PKR activated by chTBK1 (**Figure 5A**). In contrast, chIRF10 significantly inhibited the IFN-β promoter activity and the mRNA expressions of IFN-β, MX1, OASL, and PKR activated by chIKKϵ (**Figure 5B**). Similarly, chIRF10 significantly and strongly inhibited the IFN-β promoter activity and the mRNA expressions of IFN-β, MX1, OASL, and PKR activated by chIRF7 (**Figure 5C**). These results indicated that chIRF10 likely targets IRF7 to exert its regulatory role.

### chIRF10 directly targets chIRF7 to suppress its activation

**Figure 6:**
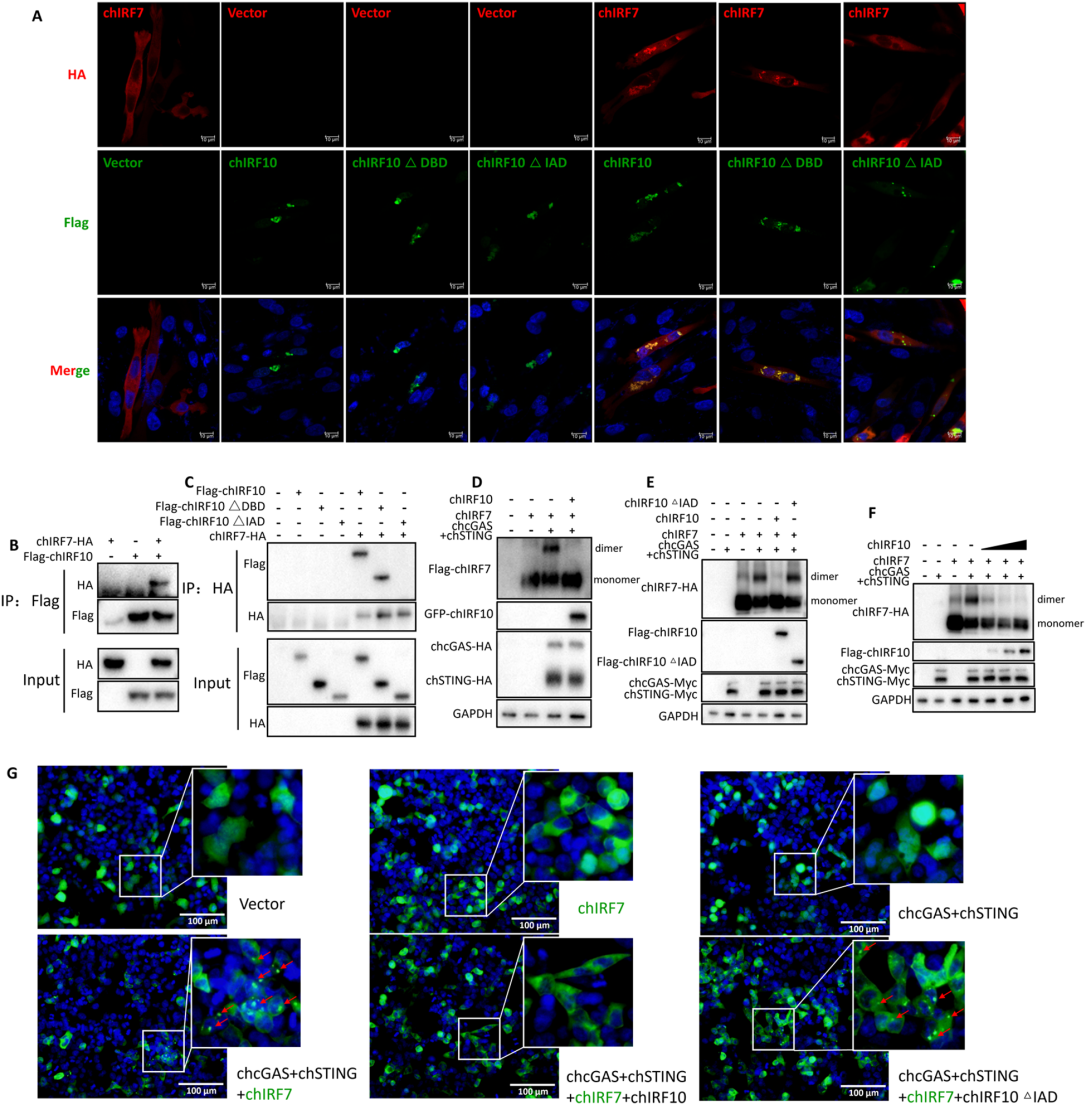
chIRF10 targets chIRF7 and inhibits chIRF7 activation. **(A)** DF-1 cells were transfected with chIRF10 and its deletion mutants, with or without chIRF7 as indicated for 24 (h) The co-localization of chIRF10/mutants and chIRF7 was examined by laser scanning confocal microscopy following IFA staining. **(B, C)** 293T cells were transfected with chIRF10 and chIRF7 **(B)**, chIRF10/chIRF10ΔDBD/chIRF10ΔIAD and chIRF7 **(C)** as indicated for 48 (h) The protein interactions between chIRF10/mutants and chIRF7 were assessed by co-IP using the indicated antibodies. **(D**–**F)** 293T cells were transfected with chIRF10, chIRF7 plus chcGAS-STING **(D)**, chIRF10/chIRF10ΔIAD, chIRF7 plus chcGAS-STING **(E)**, and increasing doses of chIRF10, chIRF7 plus chcGAS-STING **(F)** as indicated for 24 (h) The chIRF7 dimerization levels were analyzed by native PAGE. **(G)** 293T cells were co-transfected with GFP-tagged chIRF7, Flag-tagged chIRF10/chIRF10ΔIAD, and HA-tagged chcGAS-STING as indicated. The puncta formation of chIRF7 indicating its activation was observed by fluorescence microscopy 24 h post-transfection and marked as red arrows.

To explore whether chIRF10 targets IRF7 for its negative regulation, we first investigated whether chIRF10 can directly interact with the chIRF7. Confocal microscopy results revealed that both chIRF10 and chIRF10 ΔDBD exhibited significant colocalization with chIRF7, whereas the colocalization was abolished between chIRF10 ΔIAD and chIRF7 (**Figure 6A**). Similarly, co-IP assays demonstrated that chIRF10 and chIRF10 ΔDBD, but not chIRF10 ΔIAD, could strongly interact with chIRF7 (**Figures 6B, C**). Additionally, chIRF10 was also found to interact with chSTING, chTBK1, and chIKKϵ (**Supplementary Figure 1**).

Next, we examined whether chIRF10 inhibits the activation of chIRF7. The results showed that chIRF10 markedly suppressed chcGAS-STING-induced dimerization of chIRF7 (**Figure 6D**), and this inhibition was dependent on the IAD domain of chIRF10 (**Figure 6E**). Further validation indicated that chIRF10 inhibited chIRF7 dimerization in a concentration-dependent manner (**Figure 6F**). Interestingly, in 293T cells, co-transfection of chcGAS-chSTING with chIRF7 promoted the formation of distinct speckles indicated by GFP-chIRF7, which was abolished upon the addition of chIRF10 but reappeared when chIRF10 ΔIAD was introduced (**Figure 6G**). These speckles likely represent dimerized or oligomerized forms of chIRF7, indicative of its activation. In summary, consistent with the signaling activities of DBD and IAD deletion mutants of chIRF10, the results clearly demonstrated that chIRF10 interacts with chIRF7 via its IAD domain and inhibits IRF7 activation.

### chIRF10 knockout enhances the cGAMP-triggered signaling activation and suppresses viral replication in HD11 cells

**Figure 7:**
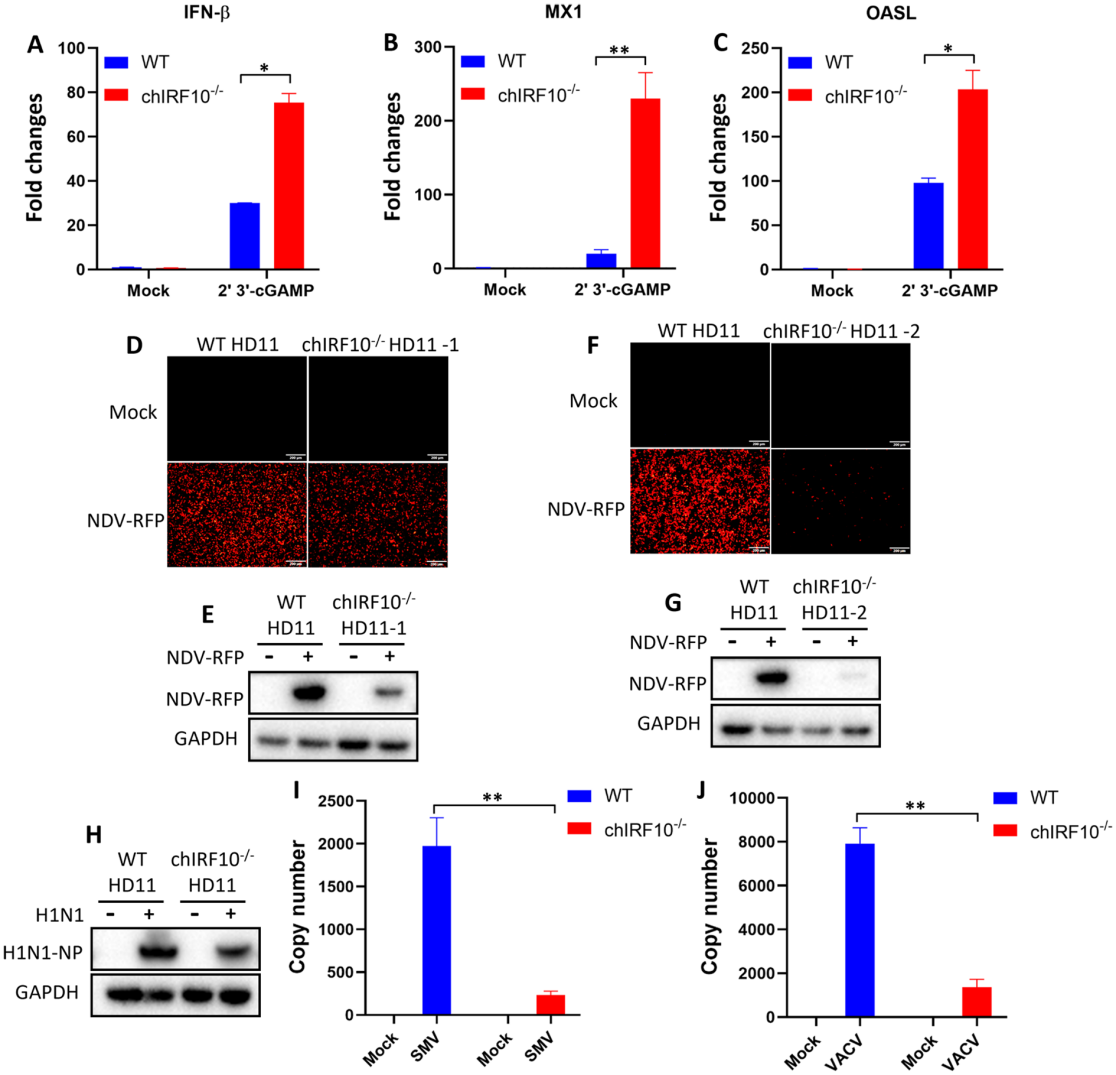
The chcGAS-STING antiviral signaling is upregulated in chIRF10^−/−^ HD11 cells. **(A**–**C)** Wild-type (WT) and chIRF10^−/−^ HD11 cells were stimulated with cGAMP for 24 h, and the mRNA expression levels of activated IFN-β **(A)**, MX1 **(B)**, and OASL **(C)** were detected by RT-qPCR. (**D**–**G)** Two independent clones of chIRF10^−/−^ HD11 cells plus WT HD11 cells were infected with NDV at 0.01 MOI for 12 (h) RFP fluorescence was observed under a fluorescence microscope **(D, F)**, and RFP protein expression was detected by WB **(E, G)**. **(H**–**J)** chIRF10^−/−^ HD11 plus WT HD11 cells were infected with AIV at 0.01 MOI **(H)**, SMV at 0.1 MOI **(I)**, or VACV at 0.1 MOI **(J)**. At 24 h post-infection, AIV NP protein expression was detected by WB, and viral copy numbers of SMV and VACV were quantified by qPCR. *p < 0.05, **p < 0.01 and ns versus controls.

To further elucidate the negative regulation of chIRF10, it was knocked out in HD11 cells and IRF10^−/−^ cells were obtained. Upon cGAMP stimulation, the absence of chIRF10 was found to enhance the mRNA expressions of cGAMP-activated IFN-β, OASL, and MX1 (**Figures 7A–C**). Secondly, two obtained chIRF10^−/−^ HD11 cell clones were infected with NDV, revealing a significant reduction in viral replication in both clones (**Figures 7D–G**). Thirdly, the replication levels of AIV (**Figure 7H**), SMV (**Figure 7I**), and VACV (**Figure 7J**) were also markedly decreased in the chIRF10^−/−^ HD11 cells. These findings further established that endogenous chIRF10 acts as a negative regulator of the cGAS-STING-IFN antiviral axis.

### Reconstitution of chIRF10 and its mutants in chIRF10^−/−^ HD11 cells re-confirms the negative regulation of chIRF10

**Figure 8:**
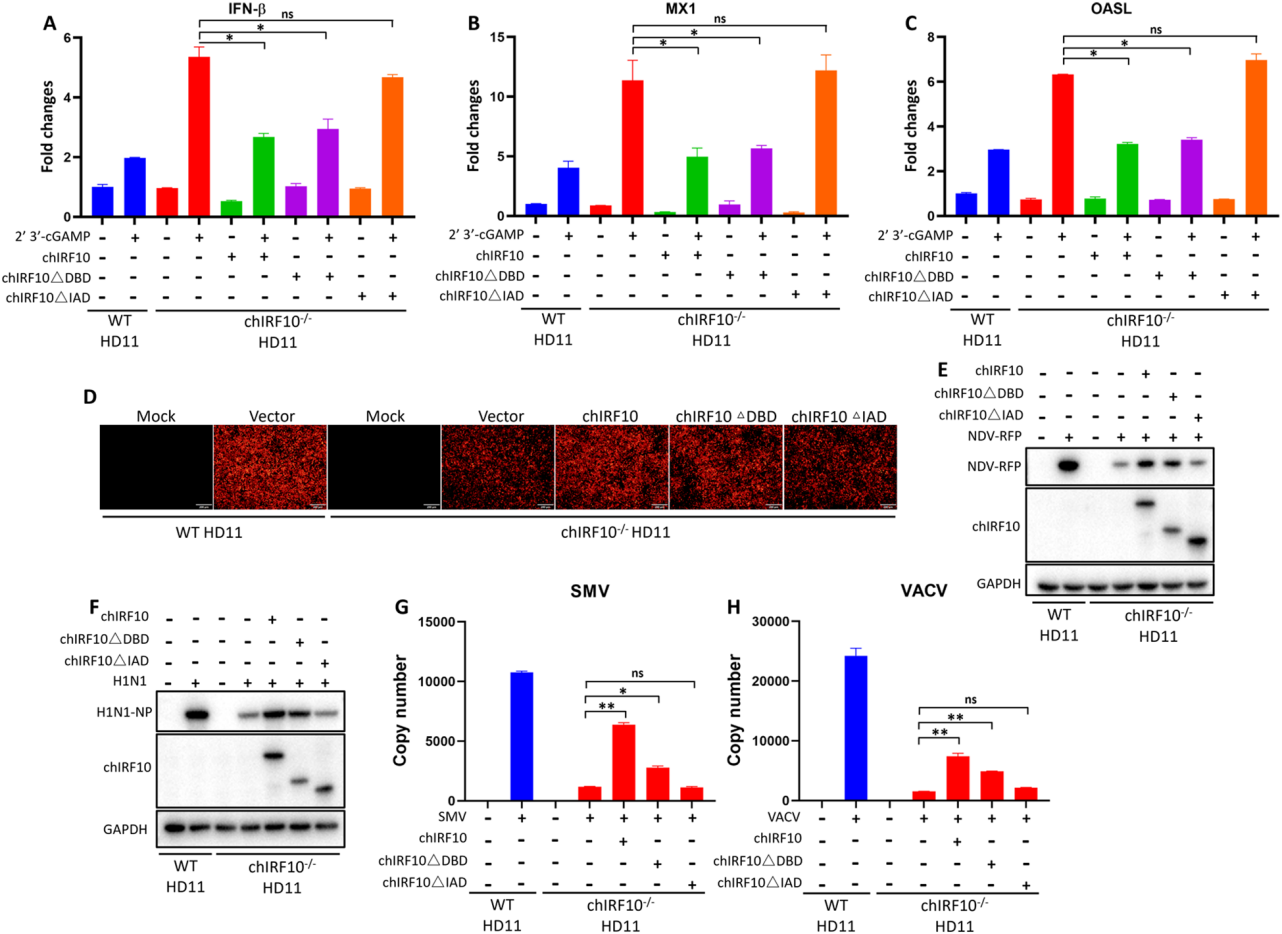
Assessment of the cGAS-STING antiviral signaling in chIRF10 reconstituted IRF10^−/−^ HD11 cells. **(A–C)** WT and reconstituted chIRF10^−/−^ HD11 cells transfected with chIRF10, chIRF10 ΔDBD, chIRF10 ΔIAD, or empty vector for 12 h were stimulated with cGAMP for 24 (h) Cells were harvested and the mRNA expression levels of IFN-β **(A)**, MX1 **(B)**, and OASL **(C)** were detected by RT-qPCR. **(D, E)** WT and reconstituted chIRF10^−/−^ HD11 cells transfected with chIRF10, chIRF10ΔDBD, chIRF10ΔIAD, or empty vector for 12 h were infected with NDV at 0.01 MOI for 12 (h) RFP fluorescence was observed by fluorescence microscopy **(D)**, and RFP protein expression was detected by WB **(E**–**H)** WT and the constituted chIRF10^−/−^ HD11 cells, as indicated, were infected with AIV at 0.01 MOI **(F)**, SMV at 0.1 MOI **(G)**, or VACV at 0.1 MOI **(H)** for 24 **(h)** The NP protein expression of AIV was detected by WB **(F)**, and viral copy numbers of SMV **(G)** and VACV **(H)** were quantified by qPCR. *p < 0.05, **p < 0.01 and ns versus controls.

It has been established that in the absence of chIRF10, cGAMP-activated IFN signaling is upregulated and viral replication is reduced. To further validate the regulation of chIRF10, chIRF10 and its mutants were reintroduced into chIRF10^−/−^ HD11 cells. First, we examined the cGAMP-activated IFN signaling after reconstitution with chIRF10, chIRF10 ΔDBD, and chIRF10 ΔIAD. Compared to wild-type (WT) HD11 cells, the mRNA levels of IFN-related genes (IFN-β, OASL, and MX1) were elevated in chIRF10^−/−^ HD11 cells upon cGAMP stimulation (**Figures 8A–C**). Notably, reconstitution with chIRF10 and chIRF10 ΔDBD, but not chIRF10 ΔIAD, reduced the expression of these cGAMP-induced IFN-related genes (**Figures 8A–C**). Second, viral replications were assessed after complementation with chIRF10 and its mutants. Relative to WT HD11, chIRF10^−/−^ HD11 cells showed decreased replications of NDV (**Figures 8D, E**) and AIV (**Figure 8F**), and all these viral replications were elevated upon reconstitution with chIRF10 or chIRF10 ΔDBD, but not with chIRF10 ΔIAD (**Figures 8D–F**). Consistent results were observed for the replication levels of SMV (**Figure 8G**) and VACV (**Figure 8H**) after reconstitution. These results further clarified the negative regulatory impact of chIRF10 on the chicken cGAS-STING-IFN antiviral signaling pathway.

## Discussion

It has been extensively demonstrated that chIRF7 acts as the primary IRF downstream of chSTING, responsible for binding to the IFN-β promoter (10, 11). However, whether other chicken IRF family members, besides chIRF7, are involved in the chicken cGAS-STING-IFN antiviral signaling pathway remains unknown. Given the limited research on chicken IRFs, this study first employed a promoter screening assay and revealed that only chIRF7 mediates type I IFN signaling triggered by chicken cGAS-STING, which is consistent with previous findings (**Figure 1A**) (10, 11). Additionally, we reported for the first time that chIRF10, which is unique to chickens, significantly negatively regulates the chicken cGAS-STING-IFN signaling pathway (**Figures 1B, C**).

Although chIRF10 was identified as early as 2002, its functional characterization has remained scarce (13, 23). Here, we unveil for the first time the negative regulatory role of chIRF10 in the chicken cGAS-STING-IFN antiviral response and further elucidate its underlying mechanism. chIRF10 potently suppresses cGAS-STING-activated IFN signaling in two chicken cell lines (DF-1 and HD11) (**Figure 2**). Our previous studies have reported the broad-spectrum antiviral activity of chicken cGAS-STING, which is further corroborated in this study (**Figure 3**) (25, 26). Further investigation into the function of chIRF10 demonstrated that it significantly inhibits the antiviral signaling activated by chicken cGAS-STING (**Figure 3**). Moreover, this study found that chIRF10 expression can be induced by viral infection (**Figure 3O**), suggesting that chIRF10 may function as an ISG and could potentially be exploited by viruses to suppress innate immune responses and facilitate immune evasion. Unfortunately, because of the lack of specific antibodies, the expression level of chIRF10 following viral infection could not be detected at the protein level.

Similar to other IRF family members, chIRF10 contains a DBD (6–116 aa) and an IAD (203–379 aa) (**Figure 4A**). This study found that it is the deletion of the IAD, rather than the DBD, that abolishes the inhibitory effect of chIRF10 on chSTING-mediated IFN activation (**Figures 4A–D**). Furthermore, the loss of the IAD also eliminates the negative regulatory effect of chIRF10 on the antiviral response triggered by chSTING (**Figures 4E–H**).

chTBK1 and chIRF7 are key kinase and transcription factors, respectively, downstream of chSTING, while IKKϵ has also been identified as an important kinase in the mammalian STING pathway (10, 28, 29). This study demonstrates that chIRF10 exerts varying degrees of inhibition on IFN signaling activated by downstream components of chSTING, including chTBK1 (**Figure 5A**), chIKKϵ (**Figure 5B**), and chIRF7 (**Figure 5C**). Intriguingly, while chIRF10 strongly inhibits chIRF7 and chIKKε, it exhibits only weak inhibition toward chTBK1, and it might suggest the potential existence of additional transcription factors downstream of chTBK1 responsible for the transcriptional activation of type I IFN. Alternatively, it could also be due to differential affinities of chIRF10 for the two kinases chIKKε and chTBK1 or their activation complexes. Despite the fact that chIRF10 exhibits multifaceted interactions with various signaling mediators, including chSTING, chTBK1, chIKKϵ, and chIRF7 (**Supplementary Figure 1**), chIRF7 was regarded as the primary target of chIRF10, given the dependence of chIRF10 inhibition on its IAD (**Figure 4**).

It is known that the IAD of IRFs mediates interactions with other IRF proteins (27). In our study, chIRF10 was found to significantly and strongly inhibit IFN signaling activated by chIRF7, and this regulatory activity is dependent on its IAD (**Figures 4**, **5**). Therefore, we investigated the interaction between chIRF10 (and its mutants) and chIRF7. We found that chIRF10 strongly interacts with chIRF7 in a manner dependent on the IAD (**Figures 6A–C**). Similar to IRF3, IRF7 forms dimers that translocate into the nucleus to initiate type I IFN transcription. This study demonstrates that chIRF10 inhibits the formation of chIRF7 dimers induced by chicken cGAS-STING, and this inhibition also requires the IAD (**Figures 6D–F**). Interestingly, we observed that chicken cGAS-STING expression in 293T cells promotes the formation of distinct punctate structures indicated by GFP-tagged chIRF7 (**Figure 6G**). We thought that this punctate pattern represents the oligomeric state of chIRF7 in the signalosome complex containing STING (30–32), indicative of its activation. Furthermore, chIRF10, but not chIRF10 ΔIAD, suppresses this punctate formation, providing additional evidence at the cellular level for the negative regulatory role of chIRF10 toward chIRF7 (**Figure 6G**).

Finally, knockout of chIRF10 in HD11 cells resulted in significantly enhanced IFN signaling activated by cGAMP, along with markedly reduced viral replication levels in the knockout cells (**Figure 7**). Following the complementation of chIRF10^−/−^ HD11 cells with WT chIRF10 or chIRF10 ΔDBD, a significant suppression of cGAMP-induced IFN signaling and a concomitant increase in viral replications were observed. In contrast, cells complemented with chIRF10 ΔIAD exhibited no such effects (**Figure 8**). These results further confirm the negative regulatory role of chIRF10 in the cGAS-STING-IFN antiviral signaling pathway.

**Figure 9:**
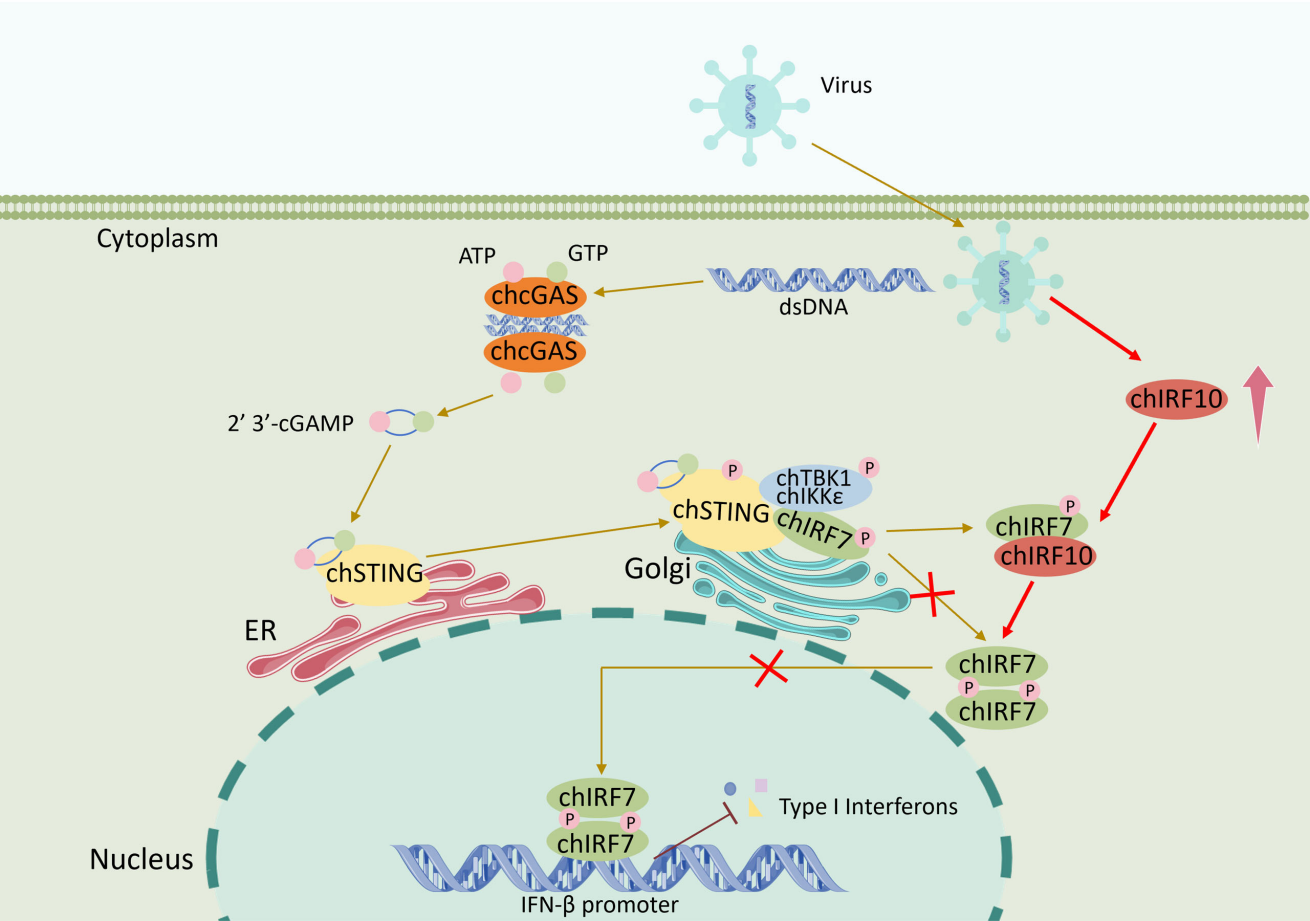
Schematic mechanism of action for the chIRF10 negative regulation of chicken cGAS-STING-IFN antiviral signaling. The invading virus directly releases its genomic dsDNA into the cytosol, or damages host cells, causing the release of mitochondria or nuclear DNA into the cytosol, where the DNA is recognized by the DNA receptor chçGAS. Upon binding dsDNA, chçGAS catalyzes ATP and GTP into the 2′3′-cGAMP, which, as a second messenger, activates chSTING on the endoplasmic reticulum (ER). Activated chSTING translocates from ER to Golgi apparatus, allowing chSTING to recruit and activate the kinases chTBK1/IKKϵ and transcription factor chIRF7. The phosphorylated chIRF7 forms a dimer, translocates from the cytoplasm into the nucleus, and binds the type I IFN promoter to initiate IFN transcription. In contrast, during viral infection, the chIRF10 is upregulated. chIRF10 interacts with chIRF7 via its IAD domain and inhibits chIRF7 activation, thereby negatively regulating the chicken cGAS-STING-IFN antiviral signaling.

In summary, we are the first to identify the negative regulatory function of chIRF10 in the cGAS-STING-IFN antiviral pathway and elucidate the underlying mechanism, in which chIRF10 targets chIRF7, interferes with chIRF7 dimerization by binding and physical sequestration, and thereby suppresses chIRF7 activation (**Figure 9**). Since chickens lack IRF3, chIRF7 acts as the sole primary transcription factor for type I IFN induction. In this context, the inhibitory role of chIRF10 is particularly critical as there is no functional redundancy between IRF7 and IRF3. Thus, chIRF10 exerts its unique role as a “master brake” in avian innate immunity, signifying the evolutionary importance.

## Data Availability

The original contributions presented in the study are included in the article/**Supplementary Material**. Further inquiries can be directed to the corresponding author.

## Supplementary material

Supplementary Figure 1 Protein Interactions between chIRF10 and chSTING, chTBK1, chIKKϵ and chIRF7. 293T cells were co-transfected with chIRF10 together with chSTING, chTBK1, chIKKϵ and chIRF7 as in the indicated combinations. At 48 h post-transfection, cells were harvested and analyzed for protein interactions by using co-IP assay.
