# Supplementary material for: Chicken IRF10 suppresses the cGAS-STING-IFN antiviral signaling pathway by targeting IRF7
Source: Front Immunol. 2026 Feb 10;17:1767491. doi: 10.3389/fimmu.2026.1767491 (PMC12929097; doi:10.3389/fimmu.2026.1767491)
Supplement: Supplementary Figure 1 — Protein Interactions between chIRF10 and chSTING, chTBK1, chIKKϵ and chIRF7. 293T cells were co-transfected with chIRF10 together with chSTING, chTBK1, chIKKϵ and chIRF7 as in the indicated combinations. At 48 h post-transfection, cells were harvested and analyzed for protein interactions by using co-IP assay. [file Supplementaryfile1.docx]

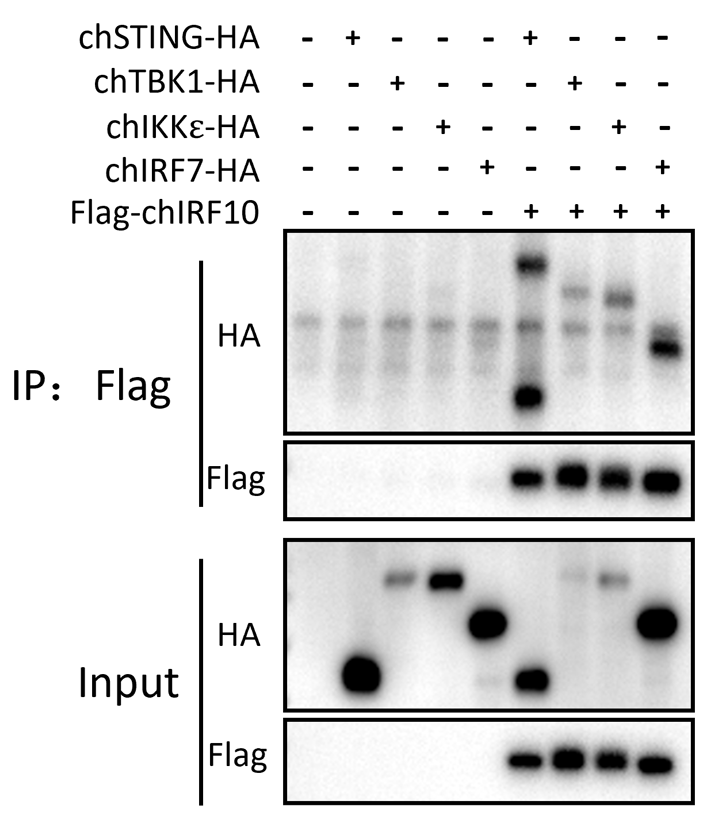


**Supplementary Figure 1.** Protein Interactions between chIRF10 and chSTING, chTBK1, chIKKε and chIRF7. 293T cells were co-transfected with chIRF10 together with chSTING, chTBK1, chIKKε and chIRF7 as in the indicated combinations. At 48 h post transfection, cells were harvested and analyzed for protein interactions by using Co-IP assay.
