# Supplementary material for: Chicken IRF10 suppresses the cGAS-STING-IFN antiviral signaling pathway by targeting IRF7
Source: Front Immunol. 2026 Feb 10;17:1767491. doi: 10.3389/fimmu.2026.1767491 (PMC12929097; doi:10.3389/fimmu.2026.1767491)
Supplement: Supplementary file 2 [file Supplementaryfile2.docx]

**Supplementary Table 1. PCR primers used for cloning and construction of chicken IRFs, TBK1 and IKKε.**

| Primer Names | Sequences (5′-3′) | |
| --- | --- | --- |
| p3xFlag-CMV-7.1-chIRF2-F | aagcttgcggccgcgaattcacctgtcgaaagaatgcggatg | |
| p3xFlag-CMV-7.1-chIRF2-R | ccgggatcctctagagtcgacttagcagctcttgactcttga | |
| p3xFlag-CMV-7.1-chIRF4-F | aagcttgcggccgcgaattcacctgtcgaaagaatgcggatg | |
| p3xFlag-CMV-7.1-chIRF4-R | ccgggatcctctagagtcgacttattcttgaatagaggaatg | |
| p3xFlag-CMV-7.1-chIRF5-F | aagcttgcggccgcgaattcagcgtccccggtgccgcgccgc | |
| p3xFlag-CMV-7.1-chIRF5-R | ccgggatcctctagagtcgactcacagcgggggccgtgggcc | |
| p3xFlag-CMV-7.1-chIRF6-F | aagcttgcggccgcgaattcagcgttacacccgcgcagggtg | |
| p3xFlag-CMV-7.1-chIRF6-R | ccgggatcctctagagtcgactcactgcgcaggcagtggctg | |
| p3xFlag-CMV-7.1-chIRF8-F | aagcttgcggccgcgaattcatgtgaccgcaatggtggcaga | |
| p3xFlag-CMV-7.1-chIRF8-R | ccgggatcctctagagtcgactcacacggcgatctgctggtt | |
| p3xFlag-CMV-7.1-chIRF10-F | aagcttgcggccgcgaattcagcggagccggggtctcccatg | |
| p3xFlag-CMV-7.1-chIRF10-R | ccgggatcctctagagtcgactcagggctggcagagctgctg | |
| pEGFP-C1-chIRF2-F | tacaagtccggactcagatctcctgtcgaaagaatgcggatg | |
| pEGFP-C1-chIRF2-R | ggatcccgggcccgcggtaccttagcagctcttgactcttga | |
| pEGFP-C1-chIRF4-F | tacaagtccggactcagatctaacttggagccgggtgagtgc | |
| pEGFP-C1-chIRF4-R | ggatcccgggcccgcggtaccttattcttgaatagaggaatg | |
| pEGFP-C1-chIRF5-F | tacaagtccggactcagatctgcgtccccggtgccgcgccgc | |
| pEGFP-C1-chIRF5-R | ggatcccgggcccgcggtacctcacagcgggggccgtgggcc | |
| pEGFP-C1-chIRF6-F | tacaagtccggactcagatctgcgttacacccgcgcagggtg | |
| pEGFP-C1-chIRF6-R | ggatcccgggcccgcggtacctcactgcgcaggcagtggctg | |
| pEGFP-C1-chIRF8-F | tacaagtccggactcagatcttgtgaccgcaatggtggcaga | |
| pEGFP-C1-chIRF8-R | ggatcccgggcccgcggtacctcacacggcgatctgctggtt | |
| pEGFP-C1-chIRF10-F | tacaagtccggactcagatctgcggagccggggtctcccatg | |
| pEGFP-C1-chIRF10-R | ggatcccgggcccgcggtacctcagggctggcagagctgctg | |
| pCAGGS-chTBK1-2HA-F | CATCATTTTGGCAAAGAATTCatgcagagcacctcgaattac | |
| pCAGGS-chTBK1-2HA-R | | GTATGGGTAGCTGGTgatatcgatacagtccacattcctcag |
| pCAGGS-chIKKε-2HA-F | CATCATTTTGGCAAAGAATTCatgcagagcacccccaactac | |
| pCAGGS-chIKKε-2HA-R | GTATGGGTAGCTGGTgatatcgaccccaggagcaggagcagc | |

**Supplementary Table 2. PCR primers used for the construction of chIRF10 mutants.**

| Primer Names | Sequences (5′-3′) |
| --- | --- |
| p3xFlag-CMV-7.1-chIRF10 △DBD-F | tcccgagtgccccccggctccg |
| p3xFlag-CMV-7.1-chIRF10 △DBD-R | cggagccggggggcactcggga |
| p3xFlag-CMV-7.1-chIRF10 △IAD-F | cgaggatgtcaatcacttccaccacgcccagc |
| p3xFlag-CMV-7.1-chIRF10 △IAD-R | gctgggcgtggtggaagtgattgacatcctcg |

**Supplementary Table 3. Primers used for qPCR in this study.**

| Primer Names | Sequences (5′-3′) |
| --- | --- |
| chGAPDH-F | agggtggtgctaagcgtgttat |
| chGAPDH-R | cagcagccttcactaccctctt |
| chIFN-β-F | atcttcgtcaccaggatgccaa |
| chIFN-β-R | cgtgccttggtttacgaagcat |
| chMX1-F | AATAAGGCTACTATCCCACA |
| chMX1-R | GTGTACTTTTGGAGTTCCTT |
| chOASL-F | ctgtccttcggagtcagcatca |
| chOASL-R | tcagcagctccagtgcatactt |
| chPKR-F | ATCTCCTCTACCTGCGGATG |
| chPKR-R | GGGTCTCCGGTACGGTTTAT |
| SMV/VACV-F | TCTGATGTTGTTGTTCGCTGCT |
| SMV/VACV-R | TCCATCTCCCTCTGGACCGCAT |

**Supplementary Table 4. The CRISPR gRNA encoding DNA sequences and PCR primers for detection of chIRF10 gene knockout.**

| gRNA Names | Sequences (5′-3′) |
| --- | --- |
| chIRF10-gRNA1-F | CACCGctgatcgcgcagatcgacag |
| chIRF10-gRNA1-R | aaacctgtcgatctgcgcgatcagC |
| chIRF10-gRNA2-F | CACCGtgcggaagagcgtccgctcg |
| chIRF10-gRNA2-R | aaaccgagcggacgctcttccgcaC |

| PCR Primer Names | Sequences (5′-3′) |
| --- | --- |
| chIRF10 gRNAdetection-F | cgctttcgctttcagggctgc |
| chIRF10 gRNAdetection-R | ggatgtagggggagtgagcgc |
